# Simulation of Dry Matter Production and N Uptake in Processing Pepper and Broccoli with the VegSyst Model Adapted to Outdoor Conditions

**DOI:** 10.3390/plants15131934

**Published:** 2026-06-23

**Authors:** José María Vadillo, Carlos Campillo, Marisa Gallardo, Sandra Millán, Henar Prieto

**Affiliations:** 1 Department of Horticulture, Center for Scientific and Technological Research of Extremadura (CICYTEX), Finca La Orden, Gobierno de Extremadura, Autovía A-V, km 372, Guadajira, 06187 Badajoz, Spain; carlos.campillo@juntaex.es (C.C.); sandra.millan@juntaex.es (S.M.); henar.prieto@juntaex.es (H.P.); 2 Department of Agronomy, University of Almeria, Ctra. de Sacramento s/n, La Cañada de San Urbano, 04120 Almería, Spain; mgallard@ual.es

**Keywords:** crop growth, nitrogen uptake, outdoor vegetables, simulation

## Abstract

Horticultural intensification in Mediterranean areas has increased the risk of nitrate pollution due to inefficient irrigation and nitrogen fertilisation management. The availability of simulation models aimed at rational nitrogen management in outdoor crops is limited. The objective of this study is to adapt the VegSyst model, initially developed for greenhouse vegetables, for use in open-field conditions in relevant crops, such as processing peppers and broccoli in Extremadura. VegSyst simulates dry matter production and nitrogen uptake by incorporating the influence of evaporative demand (TUE approach) in addition to the effect of radiation (RUE approach). Experimental field data obtained in five campaigns (peppers: 2020–2022; broccoli: 2020 and 2022) under different nitrogen doses were used. The model was calibrated, and critical N dilution curves were developed for each crop. Subsequently, the simulation of fi-PAR, dry matter production (DMP) and N uptake was validated using statistical indices (RMSE, RE, d, EF) and regression analysis. The model showed a high predictive capacity for N uptake in both crops, with values of d ≥ 0.98 and EF ≥ 0.90 in the validation campaigns. The fi-PAR simulation was acceptable in peppers and excellent in broccoli. In contrast, the DMP prediction showed notable deviations in peppers, especially in 2022, attributable to interannual variations in weather conditions and physiological limitations not considered by the model. In both crops, the TUE-based strategy was a better fit for the measurements than the RUE-based strategy, indicating that under semi-arid Mediterranean conditions, transpiration is the limiting factor for biomass production. The adaptation of the VegSyst-Outdoors model proved to be robust for simulating N uptake and sufficiently accurate to be integrated into decision support tools aimed at efficient fertilisation and irrigation management.

## 1. Introduction

There are several factors that cause nitrate pollution of groundwater and the degradation of its quality, but in areas of intensive horticulture, over-fertilisation plays a major role. The high rates applied by producers and the low nitrogen (N) uptake efficiency of horticulture crops [1,2], combined with incorrect irrigation management [3], lead to the leaching of nutrients, mainly N, into groundwater bodies. Excess or incorrect application of N fertilisers also increases greenhouse gas emissions [4].

Open-field vegetable production systems are particularly prone to contaminating surrounding water bodies [5]. In Las Vegas del Guadiana (Extremadura, Spain), where open-field horticulture is one of the most important agricultural systems, nitrate pollution is increasing. This region has been declared a nitrate-vulnerable zone (NVZ) under the EU Nitrate Directive [6] and farmers are required to improve N and irrigation management in order to reduce NO_3_^−^ leaching losses [7]. Although agriculture is not solely responsible for this situation, one of the measures that was recently approved to combat this problem is a regional law that requires growers in the area to improve their irrigation scheduling by using scientific approaches such as the use of soil water content sensors, with their use even being mandatory for large plots [8]. This problem is spreading across Europe at an alarming rate. The number of areas contaminated by nitrates has increased in recent years [6]. The same issue exists globally in areas where agricultural production and other industries responsible for this pollution are intensifying.

A key measure to reduce nitrate leaching or runoff-induced groundwater pollution is to clearly define the crop irrigation and N requirements [9]. This is a challenging endeavour, as the objective is to maintain optimal plant nutrition and thereby enable higher yields. Plants must have access to and be able to absorb the N available in the environment to meet their needs throughout the crop cycle. Under field conditions, both the availability and the uptake capacity of the plant vary with time and agro-meteorological conditions, making it difficult to determine appropriate fertiliser rates. Simplified methods based on an input–output balance are available, such as Fertilicalc [10].

A more advanced method involves the use of crop models that simulate N uptake over the cycle as a function of crop development, soil availability, etc. A crop simulation model is a mathematical and computational tool that represents, in a simplified way, the manner in which an agricultural system functions. The primary objective of a crop simulation model is to simulate or predict the behaviour of a crop under different conditions, including climate, soil, farm management and irrigation practices. These models have the potential to serve as the conceptual foundation for the development of decision support systems (DSSs), which are tools that integrate information and models to facilitate informed decision making. A DSS employs data, simulation models and other informational sources to recommend specific actions aimed at optimising crop management. Adapting and implementing these DSSs means improving aspects such as irrigation [11], fertilisation [12], and pest control [13], among others. Currently, only a limited number of simulation models are available for the efficient fertilisation management of vegetable crops. These include EU-Rotate_N [14], RB209, Azofert [15], and N-Expert 4, as outlined in the review by Gallardo et al. (2020) [16].

The VegSyst-DSS is based on the VegSyst simulation model [17,18]. Initially, the model was used to simulate crop growth, N uptake and crop evapotranspiration (ETc) in greenhouse vegetable crops such as processing pepper [19], tomato [17], muskmelon [20], melon, zucchini and cucumber [21]. Subsequently, the model was expanded to include macronutrient uptakes [22]. The implementation of this model has also been studied in other areas of the world, including Uruguay for greenhouse tomato crops [23,24]. Giménez et al. (2019) [25] adapted the VegSyst model to outdoor conditions for leafy vegetables and processing tomato.

The objectives of this work, related to implementation of the VegSyst model for processing pepper and broccoli under outdoor conditions, are: (i) to adapt the VegSyst model to outdoor conditions in these crops by considering the effect of the evaporative demand on daily dry matter production (DMP), in addition to the effect of radiation; (ii) to calibrate the VegSyst-Outdoors model for processing pepper and broccoli; and (iii) to validate the performance of the VegSyst-Outdoors model in simulating DMP and N uptake in these two crops.

## 2. Results

### 2.1. Model Calibration

Table 1 presents the data for model calibration of both crops. The base temperature was 10 °C for processing pepper [26] and 0 °C for broccoli [27]. The data for kc _ini_, kc _max_ and kc _end_ were those calculated according to FAO56.

The RUE values for processing pepper and broccoli were 2.1 and 3.0 g MJ^−1^ PAR, respectively. The TUE values were 6.40 g kg^−1^ for processing pepper and 7.5 g kg^−1^ for broccoli.

Finally, the relationship between N and DMP was described by CNC Equation (3), which, considering the parameters *a* and *b* (see Table 1), left the below final equations.

For processing pepper:

$$\%N\;crit=4.52\times {DMP}^{-0.392}$$

For broccoli:

$$\%N\;crit=4.51\times {DMP}^{-0.083}$$

Parameter *a* is very similar in both crops (4.52 in processing pepper and 4.51 in broccoli), but the difference is bigger in parameter *b* (−0.392 in processing pepper and −0.083 in broccoli). These equations were fitted to the data from the three processing pepper seasons (2020, 2021 and 2022) [28] and the two broccoli seasons (2020 and 2022).

### 2.2. Model Validation

#### 2.2.1. fi-PAR

**Figure 1:**
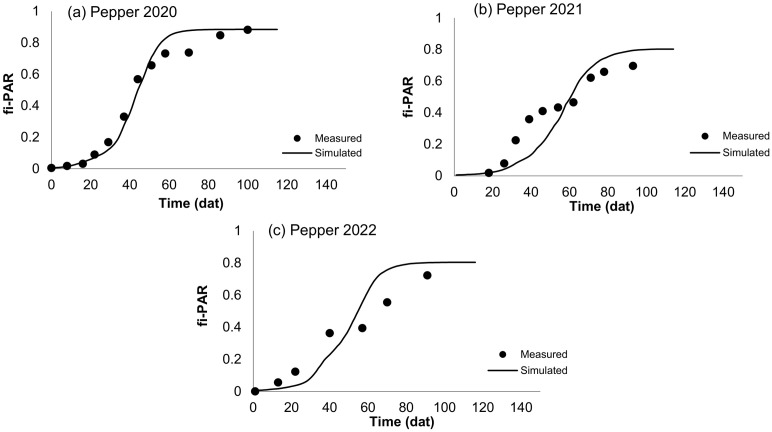
Evolution of measured and simulated processing pepper fraction of intercepted PAR (fi-PAR) data. Simulated (solid line) and measured (symbols) fi-PAR seasonal evolution as a function of the days after transplant (dat) for processing pepper in (**a**) 2020, (**b**) 2021 and (**c**) 2022.

The measured fi-PAR values for processing pepper in the calibration crop (2020) (Figure 1) fit the fi-PAR simulation curve quite well. However, in 2021 there is a more pronounced increase in fi-PAR measurements until they reach a stabilisation phase between 40 and 60 days after transplant (dat) and then continue to rise before stabilising again at the end of the crop cycle, which does not fit the simulation very well. A similar behaviour is observed in 2022, with the first stabilisation at around 40 to 57 dat, followed by a progressive increase to harvest. In the last two years, there is a decrease in fi-PAR to around 0.7 in both years, compared to 0.88 in 2020.

**Figure 2:**
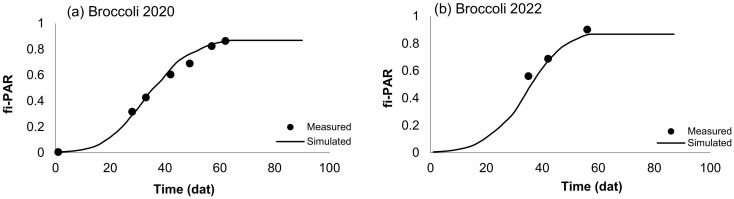
Evolution of measured and simulated broccoli fraction of intercepted PAR (fi-PAR) data. Simulated (solid line) and measured (symbols) fraction of intercepted PAR (fi-PAR) seasonal evolution as a function of the days after transplant (dat) for broccoli in (**a**) 2020 and (**b**) 2022.

In broccoli, the seasonal evolution of fi-PAR for both calibration (Figure 2a) and validation (Figure 2b) demonstrated a strong correlation between measured and simulated fi-PAR values. However, the measured values did not reach the stabilisation phase (60 dat approximately) at the end of the cycle because the crop was harvested at flowering, which prevented the fi-PAR from stabilising. The trend of the simulated values is similar to that of processing pepper, but its development is much faster, reaching maximum development earlier.

#### 2.2.2. DMP

**Figure 3:**
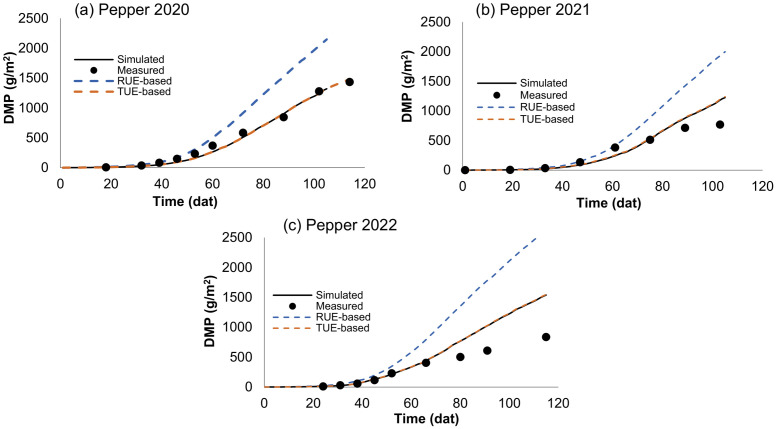
Evolution of measured and simulated processing pepper DMP data. Time course, in relation to the days after transplant (dat), of simulated and measured DMP in processing pepper in (**a**) 2020, (**b**) 2021, and (**c**) 2022.

Figure 3 shows, for each of the three processing pepper crops, the seasonal evolution of: (i) measured DMP; (ii) simulated DMP using the RUE and TUE strategies; and (iii) the final simulation calculated by the model using both strategies (grey solid line). In 2020 (Figure 3a), a perfect fit of the measurements against the final simulation is observed, because these are the data used for the calibration. In the subsequent two years, the daily simulations conducted using the model based on the TUE demonstrated a higher degree of alignment with actual data compared to the RUE. Both models exhibited an overestimation of biomass production in 2021 and 2022, with an escalating discrepancy as the harvest progressed and more pronounced differences observed in 2022.

**Figure 4:**
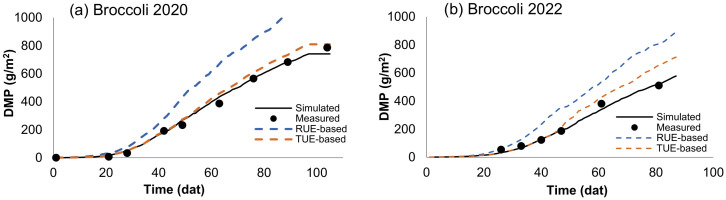
Evolution of measured and simulated broccoli DMP data. Time course, in relation to the days after transplant (dat), of simulated obtained through RUE (RUE-based) and TUE (TUE-based) and measured DMP in broccoli in (**a**) 2020 and (**b**) 2022.

In broccoli (Figure 4), both approaches demonstrated a good correlation between simulated and measured values that was stronger than that observed in processing pepper. The measured values were found to be slightly more aligned with the TUE strategy than with the final values simulated by the model in the 2020 campaign (calibration). A satisfactory fit was achieved for the validation season (2022), employing the TUE strategy, with the measured data closely matching the simulated values. It should be noted that the DMP obtained through RUE was overestimated.

#### 2.2.3. N Uptake

**Figure 5:**
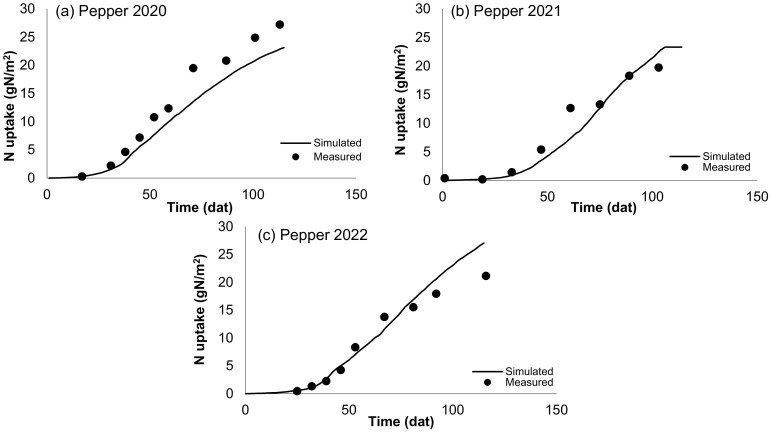
Evolution of measured and simulated processing pepper N uptake data. Time course, in relation to the days after transplant (dat), of simulated and measured N uptake in processing pepper in (**a**) 2020, (**b**) 2021, and (**c**) 2022.

Figure 5 presents the measured and simulated values of N uptake for the three processing pepper crops. In 2020, the model underestimates N uptake (Figure 5a), with measured values being higher than the simulated values from 45 dat onwards. However, in 2021 the model fits quite well with the measured values, except for 61 dat, which is the only data point that deviates slightly above the simulation (Figure 5b). In 2022, the model also fits the measured values well (Figure 5c), except for the last data point where the model gives a higher result. Higher final N uptake is observed in 2020 (27.2 g N/m^2^) than in 2021 and 2022 (19.7 and 21.1 g N/m^2^, respectively).

**Figure 6:**
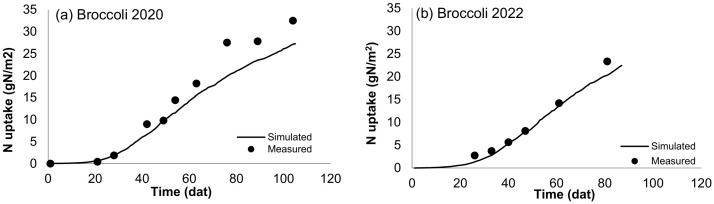
Evolution of measured and simulated broccoli N uptake data. Time course, in relation to the days after transplant (dat), of simulated and measured N uptake in broccoli in (**a**) 2020 and (**b**) 2022.

In broccoli, there was a good agreement between measured and simulated values of N uptake in the first part of the season in 2020 until approximately 63 dat (Figure 6a). From this moment onwards the model underestimated N uptake, reaching values 16% higher than the model at the end of season. In the 2022 season, there was excellent agreement between measured and simulated N uptake values throughout the cropping cycle (Figure 6b). The N uptake was higher in 2020, with a 28% lower final N uptake in 2022 compared to 2020.

#### 2.2.4. Statistical Evaluation of Model Fit

A comparison was made between measured and simulated values of DMP, N uptake and fi-PAR from the validation experiments. Table 2 summarises the results of the statistical indices used to evaluate the performance of the model in simulating cumulative values in both crops.

**Figure 7:**
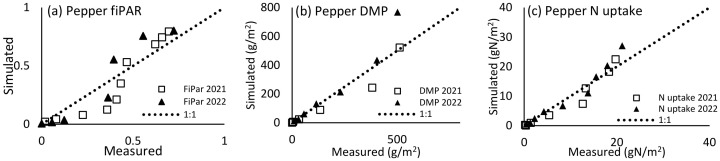
Simulated vs. measured values of (**a**) fi-PAR, (**b**) DMP, and (**c**) N uptake for processing pepper. The 1:1 line is shown in each graph.

The results of the simulation for fi-PAR in the processing pepper crop show a low RMSE of 0.12 in both years, indicating that the model accurately represents the fraction of intercepted radiation. Furthermore, the R^2^ (0.93–0.95) and d values (0.94–0.96) indicate a strong correlation between the simulated and measured values. The regression slope (m) varies between 0.98 and 1.15, which means that the model manages to capture the general trend, with a slight overestimation in 2022. The DMP prediction shows greater variability. The RMSE is notably high (165.7 g/m^2^ in 2021 and 286.8 g/m^2^ in 2022), indicating a significant deviation between measured and simulated values. Even though the Willmott index (d) is high (0.87–0.95), the efficiency of the model (EF) in 2022 is negative (−0.07), indicating that the simulation does not improve the prediction compared to a simple average of the observed data. However, Figure 7b shows that the slope, except for the last point for 2022, is very close to the 1-1 line, which may indicate that the last sample had a significant influence on the results of the indices. For N uptake, the statistical indicators reflect a high estimation capacity of the model with precision d (0.98) and R^2^ (>0.96). EF is also high (0.91–1.0), confirming that the simulation provides a good representation of nutrient uptake.

For broccoli, the model achieves an excellent representation of fi-PAR, with a low RMSE of 0.05 and values of d and R^2^ close to 0.99, indicating an almost perfect correlation between simulated and measured values. The slope (m = 0.94) indicates a slight underestimation of the model, but within an acceptable range. The prediction of DMP in broccoli shows significantly better results than in processing pepper. With a low prediction error (RMSE = 20.4 g/m^2^), an R^2^ of 0.99 and a d value of 0.99, the model is highly accurate for this crop. The efficiency of the model (EF = 0.99) confirms that the estimates correspond almost perfectly to the measured values, which suggests that the model is particularly reliable for simulating broccoli growth. The results for N uptake are also satisfactory. With an R^2^ value of 0.99, d = 0.99 and slope of 0.90, the model captures the dynamics of nutrient uptake in broccoli with great precision and without significant deviations.

For both DMP and fi-PAR (Figure 7a,b), a poor model fit is obtained when observing the statistic. For DMP in 2021, RE = 0.52 and 2022 RE = 0.93 indicate very little correlation with the model, although the other statistics are more acceptable for 2021 (d = 0.95 and EF = 0.69). For fi-PAR, the RE index also indicates a poor fit with 0.31 and 0.38 for 2021 and 2022, respectively, although the d and EF indices would provide a better interpretation. On the other hand, in Figure 7c, there is a good fit of the model in the validation years 2021 and 2022, as interpreted by the data in Table 1, where the statistical indices are RE ≤ 0.25, d ≥ 0.98 and EF ≥ 0.90.

**Figure 8:**
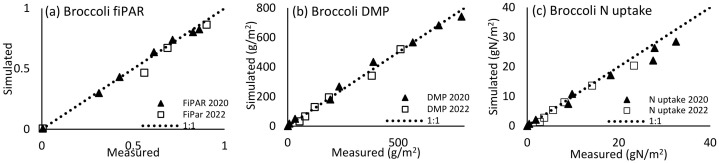
Simulated vs. measured values of (**a**) fi-PAR, (**b**) DMP and (**c**) N uptake for broccoli. The 1:1 line is shown in each graph.

Figure 8 shows the measured vs. simulated values of DMP, N uptake and fi-PAR of the 2022 broccoli validation crop. All three variables (Figure 8b,c) show a very good fit, as can also be seen in Table 2 with RE ≤ 0.15, d ≥ 0.99 and EF ≥ 0.96.

## 3. Discussion

In this study, the VegSyst model was calibrated for processing pepper and broccoli crops grown outdoors using the RUE and TUE methodologies. For both crops, the measured and simulated values are closer with the TUE than the RUE strategy. This is because the model takes data from the more limiting method on a daily basis from both strategies. Outdoors and in the environmental conditions of the area where this study was developed, the transpiration of horticultural crops is the limiting factor because, as indicated by Garruña-Hernández et al. (2014) [29], high temperatures alter stomatal conductance and reduce the biomass production capacity. This contrasts with the development of greenhouse crops, as these have reduced radiation due to the cover and their more optimal conditions, therefore favouring DMP using the RUE strategy rather than the TUE strategy because radiation is the limiting factor in their management conditions. According to Campbell and Norman (2000) [30], the RUE approach works well in humid environments where growth is limited by radiation, while the TUE approach is more suitable for drier environments where growth is limited by high VPD. When used for greenhouse crops (R^2^ ≈ 0.98 for DMP and R^2^ ≈ 0.99 for N uptake) [19], VegSyst generally results in a better model fit than for open-field crops. This is due to the more controlled conditions of different variables in the greenhouse as the crops are protected. Outdoors there are uncontrolled factors, some of which may cause interannual differences that explain deviations between the fitted data.

Another aspect that is increasingly being studied, yet little remains known about, and that complicates the interpretation of data and model calibration, is soil microbiota. Microbiota influence N uptake in horticultural crops through complex interactions that affect plant growth and N use efficiency, highlighting the importance of microbial N transformations for plant nutrition [31]. The influence of temperature on N assimilation by roots has also been studied little and, according to Le Deunff et al. (2019) [32], is a factor not considered by models. Meteorological data can also cause deviations between the fitted data, as climate conditions influence physiological processes such as flowering and fruit set or soil type, as indicated in a previous study [28].

For outdoor crops such as processing tomatoes, Giménez et al. (2019) [25] differentiated two periods to consider the RUE. This is due to the distinction between the vegetative and reproductive phases. However, for other outdoor crops such as spinach or lettuce, where the vegetative phase is the whole cycle, only one RUE is calculated, as per Giménez et al. (2019) [25]. In broccoli, the cycle is more similar to that of foliage crops, which is why only one RUE was calculated. This is because a large part of the cycle is vegetative, with the inflorescence developing only in the last few weeks. In processing pepper, we used a single period for RUE calculation because the vegetative and reproductive phases overlap and are simultaneous over most of the cycle. Other studies were conducted in conditions similar to ours (in southern Portugal) in which four RUE periods were considered according to the phenological stage of the bell pepper, and the RUE did not change substantially throughout the growing season [33]. The overlap of the vegetative and fruit set phases may explain why the DMP simulation of broccoli has a better fit than that of pepper. However, the RUE developed in this work for pepper (2.1 g MJ^−1^ PAR) is consistent with the existing literature, as Yildirim et al. (2018) [34] reported RUE values between 1.46 and 2.44 g MJ^−1^ PAR for outdoor pepper in Turkey, and Vieira et al. (2009) [33] found values of around 1.6 g MJ^−1^ PAR in Portugal.

The RUE measured in this work for broccoli (3 g MJ^−1^ PAR) is also in agreement with the RUE calculated for broccoli cultivation under Mediterranean conditions (3.2 g MJ^−1^ PAR) published by Conversa et al. (2019) [35]. However, Vågen et al. (2004) [36] reported an RUE of 2.5 g MJ^−1^ PAR for broccoli, stating that low RUE values were obtained from early plantings due to a combination of low temperature and saturated light response. Vegetable crop TUE data are very scarce, with numerous publications containing irrigation water productivity (total dry matter or crop yield/irrigation water use) data, but few with crop transpiration [37] and TUE data.

With respect to N uptake for the two studied crops in Figure 5a and Figure 6a, for the year 2020, the measured data can be seen to be above the simulated data. This is because no constraints were applied to generate the dry matter, so it is not close to the CNC, which is used to generate the model simulation from the 3 years of data. As indicated in the previous section, soil and weather conditions influenced DMP and consequently N uptake. Despite the deviations between simulated and measured data in the validation years, the best fit is that of N uptake, which is important for N fertilisation scheduling.

As shown in Figure 1a, the longer cycle length of processing pepper in 2020 resulted in a higher DMP than in the other campaigns and consequently a higher fi-PAR and more N uptake. However, the behaviour of the fi-PAR curve in 2021 and 2022 does not have the same shape as in 2020, as the last two study years have a stabilisation phase. This stabilisation shows a clear limit to growth, which can be caused by several factors. The VegSyst model simulates optimal crop growth for a set of weather conditions. If additional limiting factors are present, a discrepancy between simulated and measured values can be expected. VegSyst is a simplified model that aims to provide the technician with a calculation tool for designing a fertilisation plan. This simplicity means that it does not consider complex physiological aspects such as the effect of the soil, the presence of micro-organisms or the factors limiting flowering and fruit set in a particular crop.

Comparing the statistical validation indices of this work for outdoor pepper with those of Giménez et al. (2013) [19] for greenhouse pepper, it can be seen that the model performed better for outdoor than greenhouse pepper. This is due to the previously mentioned controlled conditions in the greenhouse. The validation data for both crops in this work were obtained from trials carried out in Extremadura, so it would be interesting to add validation data from other locations or varieties with a similar cycle to make the model more robust. In processing tomato, a process similar to the one followed in this work has been carried out. After the initial development of VegSyst for fresh tomato varieties grown in greenhouses [17], calibration and validation were subsequently carried out for processing varieties grown under outdoor conditions, such as by Giménez et al. (2019) [25].

As reported by Giménez et al. (2019) [25], EU-Rotate_N and CropSyst are complex mechanistic models which are mainly used for scenario analysis. They are not designed to be part of a DSS to support N and irrigation management. In contrast, VegSyst-Outdoors fulfils this function, as demonstrated in greenhouses [18]. This is based on a study by Suárez-Rey et al. (2016) [38] which compared the EU-Rotate and CropSyst models. Therefore, after calibrating these crops with the VegSyst model, they will be included in the DSS of irrigation and N fertilisation recommendations for the most important outdoor horticultural crops in Spain, called VegSyst-DSS Suite. This DSS will be divided into outdoor and greenhouse crops, with the latter DSS developed by Gallardo et al. (2016) [21] for the most relevant crops in southeastern Spain.

## 4. Materials and Methods

### 4.1. Model Description

The VegSyst model, initially developed for greenhouse vegetable crops by Gallardo et al. (2011; 2014; 2016) [17,20,21] and subsequently adapted to outdoor vegetable crops by Giménez et al. (2019) [39], calculates daily the fraction of photosynthetically active radiation intercepted by the crop (fi-PAR), DMP and N crop uptake during the cropping cycle. The N crop and fi-PAR measurements were carried out in accordance with the procedures described by Vadillo et al. (2024) [28]. For outdoor crops, following Gimenez et al. (2019) [25], DMP was estimated by taking the most limiting daily values from two methodologies whose Equations (1) and (2) are presented below:

(1) $$RUE=\frac{{DMP}_{i}}{{PAR}_{i}}$$

where RUE (g MJ^−1^) is radiation use efficiency, DMP_i_ (g m^−2^) is daily dry matter production and PAR_i_ (MJ m^−2^) is daily intercepted photosynthetically active radiation.

(2) $${DMP}_{i}=T_{i}\times TUE\times {VPD}^{-b}$$

where TUE (g kg^−1^) is transpiration use efficiency, b is a constant, VPD is the daily average vapour pressure deficit, and T_i_ is the daily transpiration (mm d^−1^).

The critical nitrogen curve (CNC) was determined using Equation (3) following Greenwood et al. (1990) [40]. For each biomass sampling date, an analysis of variance determined the minimum total crop N content (%N) associated with the maximum total DMP for the four tested treatments. When maximum total DMP occurred in two N treatments, the one with the lower total crop N content was selected to achieve the highest nitrogen use efficiency. Using these procedures, the critical N content (%N crit) was identified for each biomass sampling date.

(3) $$\%N\;crit=a\times {DMP}^{-b}$$

where the coefficient a is the total crop N concentration when DMP is 1 t ha^−1^ [40,41], and b is a dimensionless statistical parameter governing the slope of the relationship.

The CNC used for outdoor processing pepper was that developed by Vadillo et al. (2024) [28] and the CNC used for broccoli was that developed by Vadillo (2025) [42].

Finally, N uptake was obtained from Equation (4):

(4) N uptake=%N×DMP

**Figure 9:**
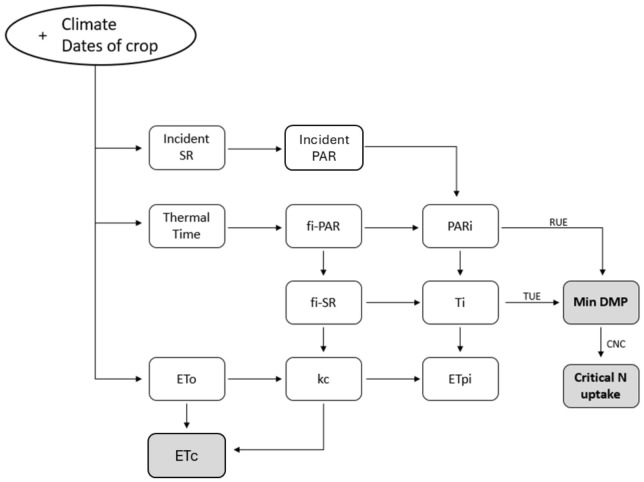
Schematic representation of the VegSyst-Outdoors simulation model. The parameters in the circle are the inputs, those in the rectangles are intermediate calculations, and those in shaded rectangles are the outputs of the model. For a description of the symbols and parameters, see Table 3.

Figure 9 shows a simplified schematic representation of the internal calculations performed by the model and the inputs required. By providing the climatic data and crop dates, we obtain for these calculations the DMP, N uptake and ETc. As illustrated in the diagram, DMP can be obtained using either the RUE or the TUE approach.

The symbols, units and names, as well as the description of the parameters used in the calibration of the VegSyst-Outdoors model [25], are represented in Table 3.

#### Model Calibration and Validation

The parameters required for the calibration and validation of the VegSyst-Outdoors model were obtained from data of open-field experiments of processing pepper and broccoli crops at the same location in different years. The model simulates the seasonal DMP of the aerial part of these crops and the N uptake. Table 4 includes information about the experiments used for calibration and for validation.

### 4.2. Experimental Site

#### 4.2.1. Processing Pepper

Three outdoor processing pepper (cv. *Ramonete lamuyo*) field experiments were carried out in 2020, 2021 and 2022 at the experimental farm ‘La Orden’, Centro de Investigación Científica y Tecnológica de Extremadura (Junta de Extremadura, Badajoz, Spain) (38°53′ N, 6°50′ W, 185 m altitude). A fertigation system was used, placing the irrigation line in the middle of two paired rows. The field experiments were arranged into 4 treatments with 4 replications in randomised blocks in 2020 and 2021. The different N doses were 0, 60, 120 and 180 kg N/ha. In 2022, there were 3 treatments (0, 200 and 300 kg N/ha) with 4 replications. Each single plot measured 12 × 6 m and included 6 beds with two rows of plants at a density of 33,000 plants/ha. This trial formed part of a broader study. Consequently, the treatment with the lowest N input that did not result in a decline in biomass production was employed on an annual basis. A detailed description of these experiments can be found in Vadillo et al. (2024) [28]. Four plants were sampled from the central beds approximately every two weeks. In these samplings, DMP, N content and fi-PAR measurements were taken for model development.

#### 4.2.2. Broccoli

Two field experiments with broccoli (cv. Parthenom) were undertaken at the same experimental farm as that for outdoor processing pepper (see Section 2.2.1) in the 2020 and 2022 seasons. Each individual plot measured 12 × 6 m and included 6 beds with two rows of plants at a density of 33,000 plants/ha. A fertigation system was used, with the irrigation line placed in the middle of the paired rows. N doses were 0, 100, 200 and 300 kg N/ha for 2020 and 0, 200 and 300 kg N/ha for 2022, with 4 replications. For each sampling date, the treatment with the optimal nutritional status, which would not have had a statistically lower DMP than the lowest N fertiliser application, was selected for the development of the model, as in the processing pepper experiments. Samples were taken every two weeks, and the same measurements were made as in the processing pepper trial. Table 4 shows the information on the two broccoli trials, and which crop was used for calibration and which for model validation.

### 4.3. Model Accuracy Evaluation

To evaluate the agreement between simulated and observed values, the statistical indices applied were: (i) root mean square error (RMSE); (ii) relative error (RE) [43]; (iii) Willmott index of agreement (d) [44]; (iv) model efficiency (EF) [45]; and (v) linear regression analysis between simulated and measured values. Values of RE ≤ 0.25, d ≥ 0.75 and EF ≥ 0.6 were considered to indicate good performance of the model, following Yang et al. (2014) [46]. The simulated and measured data were fitted to a linear regression by comparing the slope and coefficient of determination (R^2^) to the baseline 1:1 using confidence interval tests.

## 5. Conclusions

In this study, the VegSyst model was calibrated and validated for two very different vegetable crops, processing pepper and broccoli, in outdoor conditions. In the case of processing pepper, a satisfactory fit was achieved between simulated and measured data following calibration with 2020 processing pepper data. However, in the validation of 2021 and 2022 processing pepper, a substantial deviation was observed between the simulated and measured fi-PAR and DMP data. Conversely, calibrating the model for broccoli yielded a favourable fit between the simulated and measured data for fi-PAR and DMP.

In outdoor vegetable crops in southwest Spain and under environmental conditions similar to those of this location, transpiration limits biomass production due to high temperatures, affecting stomatal conductance and producing a yield-limiting effect. This makes the results in field crops closer when using the TUE strategy than the RUE strategy. The opposite is true for greenhouse crops, where controlled conditions favour higher dry matter production with the RUE strategy. However, biomass production is limited by the RUE strategy due to lower canopy radiation.

In both crops, the nitrogen uptake simulation closely approximated the experimental measurements, suggesting its potential as a reliable foundation for adjusting the calculation and seasonal distribution of nitrogen in fertiliser programmes. The simplicity of the model and its good fit for the crops studied make it a sound option for incorporation into decision support systems to improve the irrigation and fertilisation management undertaken by growers.

## Figures and Tables

**Table 1:** Calibration parameters for processing pepper and broccoli in the VegSyst-Outdoors model.

Crop Growth Parameters	Processing Pepper	Broccoli
T base (°C)	10	0
f _0_	0.005	0.005
f _f_	0.880	0.910
f _mat_	0.880	0.999
RTT _0.5_	0.348	0.640
GDD_f_ (°C/day)	1522	990
GDD_mat_ (°C/day)	1715	990
RUE (g MJ^−1^ PAR)	2.1	3.0
TUE (g kg^−1^)	6.40	7.5
Water use parameters		
kc _ini_	0.30	0.40
kc _max_	1.10	1.15
kc _end_	1.10	0.70
Critical N curve (CNC) parameters		
*a*	4.52	4.51
*b*	−0.392	−0.083

**Table 2:** Statistical indices of the validation for processing pepper and broccoli of the VegSyst-Outdoors model.

Parameter	Crop	n	RMSE	RE	d	EF	Slope (m)	R^2^
fi-PAR	Pepper 2021	10	0.12	0.31	0.94	0.69	0.98	0.93
	Pepper 2022	7	0.12	0.38	0.96	0.77	1.15	0.95
	Broccoli 2022	3	0.05	0.09	0.99	0.98	0.94	0.99
DMP	Pepper 2021	8	165.7	0.52	0.95	0.69	1.25	0.95
	Pepper 2022	9	286.8	0.93	0.87	−0.077	1.63	0.97
	Broccoli 2022	6	20.4	0.09	0.99	0.99	0.98	0.99
N uptake	Pepper 2021	8	2.2	0.25	0.98	0.91	0.97	0.96
	Pepper 2022	9	2.4	0.25	0.98	0.90	1.10	0.98
	Broccoli 2022	6	1.5	0.15	0.99	0,96	0.90	0.99

RMSE: root mean square error; RE: relative error; d: Wilmott index of agreement; EF: model efficiency; m: slope of the relationship between simulated and measured values; R^2^: coefficient of determination of the linear relationship between simulated and measured values; and n is the amount of data.

**Table 3:** Symbols, units and description of parameters in the VegSyst-Outdoors model.

Symbol	Units	Description
f_i_-PAR		Fraction of photosynthetically active radiation (PAR)
PAR_i_	(MJ m^−2^ d^−1^)	Daily PAR intercepted by the crop at day i
RUE	(g MJ^−1^ PAR)	Radiation use efficiency
TUE	(g kg^−1^)	Transpiration use efficiency
f_i-SR_		Fraction of solar radiation (SR) intercepted by the crop at day i
Ti	(mm d^−1^)	Daily transpiration
k_c_		Crop coefficient
ETc	(mm d^−1^)	Daily crop evapotranspiration
ETp_i_	(mm d^−1^)	Daily potential evapotranspiration
ETo_i_	(mm d^−1^)	Daily reference evapotranspiration

**Table 4:** Summary of field experiments and use for calibration (C) and validation (V).

Crop	Year	Use	Locations	Cultivar	Transplanting Date	Date of End of Crop	Duration (Days)
Processing pepper	2020	C	Badajoz (Spain)	*Ramonete*	18 May 2020	30 September 2020	135
Processing pepper	2021	V	Badajoz (Spain)	*Ramonete*	14 May 2021	15 September 2021	124
Processing pepper	2022	V	Badajoz (Spain)	*Ramonete*	12 May 2022	6 September 2022	117
Broccoli	2020	C	Badajoz (Spain)	*Parthenom*	27 August 2020	2 December 2020	97
Broccoli	2022	V	Badajoz (Spain)	*Parthenom*	2 September 2022	28 November 2022	87

## Data Availability

Data available on request due to restrictions.
